# Interleukin-6 Knockout Inhibits Senescence of Bone Mesenchymal Stem Cells in High-Fat Diet-Induced Bone Loss

**DOI:** 10.3389/fendo.2020.622950

**Published:** 2021-02-19

**Authors:** Yujue Li, Lingyun Lu, Ying Xie, Xiang Chen, Li Tian, Yan Liang, Huifang Li, Jie Zhang, Yi Liu, Xijie Yu

**Affiliations:** ^1^ Department of Endocrinology and Metabolism, Laboratory of Endocrinology and Metabolism, Rare Disease Center, West China Hospital, Sichuan University, Chengdu, China; ^2^ Department of General Practice, West China Hospital, Sichuan University, Chengdu, China; ^3^ Department of Integrated Traditional Chinese and Western Medicine, West China Hospital, Sichuan University, Chengdu, China; ^4^ Research Core Facility, West China Hospital, Sichuan University, Chengdu, China; ^5^ Department of Rheumatology and Immunology, Rare Disease Center, West China Hospital, Sichuan University, Chengdu, China

**Keywords:** IL-6, obesity, osteoporosis, senecence, bone mesenchymal stem cells

## Abstract

Obesity, a chronic low-grade inflammatory state, not only promotes bone loss, but also accelerates cell senescence. However, little is known about the mechanisms that link obesity, bone loss, and cell senescence. Interleukin-6 (IL-6), a pivotal inflammatory mediator increased during obesity, is a candidate for promoting cell senescence and an important part of senescence-associated secretory phenotype (SASP). Here, wild type (WT) and (IL-6 KO) mice were fed with high-fat diet (HFD) for 12 weeks. The results showed IL-6 KO mice gain less weight on HFD than WT mice. HFD induced trabecular bone loss, enhanced expansion of bone marrow adipose tissue (BMAT), increased adipogenesis in bone marrow (BM), and reduced the bone formation in WT mice, but it failed to do so in IL-6 KO mice. Furthermore, IL-6 KO inhibited HFD-induced clone formation of bone marrow cells (BMCs), and expression of senescence markers (p53 and p21). IL-6 antibody inhibited the activation of STAT3 and the senescence of bone mesenchymal stem cells (BMSCs) from WT mice *in vitro*, while rescued IL-6 induced senescence of BMSCs from IL-6 KO mice through the STAT3/p53/p21 pathway. In summary, our data demonstrated that IL-6 KO may maintain the balance between osteogenesis and adipogenesis in BM, and restrain senescence of BMSCs in HFD-induced bone loss.

## Introduction

Obesity and osteoporosis are common diseases with increasing prevalence worldwide. Obesity is often accompanied by metabolic complications, including insulin resistance, type 2 diabetes (T2D) and liver steatosis (1, 2). Osteoporosis is characterized by low bone mass with microstructure disruption, resulting in skeletal fragility and increased risk of fracture (3, 4). For a long time, obesity was recognized as a condition with positive effects on bone, mainly because weight gain on bone has been considered to increase mechanical loading exerted on the skeleton, and then bone mass would increase to accommodate weight gain (5). However, some epidemiologic studies indicate that obesity is associated with increased incidence of fractures (6, 7). In general, the increase of mechanical load caused by obesity initially promotes the increase of bone mass, while the metabolic disorders caused by long-term obesity leading to the decrease of bone formation and bone turnover (8). In recent years, studies on obesity and bone metabolism have attracted academic attention. However, the mechanisms underlying the bone mass acquired in obesity remains not well known.

There are two main imbalances in the development of osteoporosis (OP): one is the uncoupled bone remodeling involving bone-forming osteoblasts and bone-resorbing osteoclasts, the other lies in bone-fat imbalance (9). Current evidence indicates that the differentiation potential of bone mesenchymal stem cells (BMSCs) is strictly regulated by the bone marrow (BM) microenvironment, including cytokines and hormones in BM (10). Changes in the BM microenvironment determine the commitment of BMSCs into the osteoblast or adipocyte lineage (11, 12). The balance between adipogenesis and osteogenesis of BMSCs would be broken when the BM microenvironment changes. The eWAT-derived cytokines may dysregulate the interaction between adipogenesis and osteogenesis of BMSCs through endocrine pathway. Bone marrow adipose tissue (BMAT) exerts influence on osteoblasts, osteoclasts and hematopoietic cells through paracrine pathway (13). Obesity causes chronic low-grade inflammatory state. The adipose tissue derived inflammatory cytokines promote obesity-related metabolic disorders. The level of inflammatory cytokine is discrepant in different adipose tissues, and visceral adipose tissue (VAT) produces more pro-inflammatory cytokines than subcutaneous adipose tissue (SAT) (14). Although the underlying mechanism is not clear, increased levels of inflammatory factors (such as TNF-α, IL-1, IL-6, and IL-17) have been traditionally thought to contribute to bone loss (15–17).

IL-6, a multifunctional cytokine, is produced by adipocytes, monocytes, endotheliocytes and hepatocytes (18). As one important mediator of chronic low-grade inflammation, the level of IL-6 is higher in VAT than SAT in mice (19). However, the level of IL-6 in BM is not exactly known. IL-6 initiates intracellular signal transduction by binding to its membrane-bound receptor IL-6Rα or its soluble receptor sIL-6R (20). Studies have shown that levels of IL-6 and sIL-6R increase during the osteogenic differentiation of BMSCs. Then, the IL-6/sIL-6R complex could activate the downstream signal transducer and activator of transcription 3 (STAT3) signaling pathway, promote the osteogenic differentiation of BMSCs through autocrine or paracrine feedback loops (21, 22). The IL-6/sIL-6R complex could enhance alkaline phosphatase (ALP) activity of human (MG-63) and murine (MC3T3-E1) osteoblastic cell lines as well as primary murine calvaria cells (23, 24). However, the activation of the gp130 signaling pathway by the IL-6/sIL-6R complex was initially thought to regulate osteoclast formation in bone (25). Early research also suggested that IL-6 inhibited bone nodule formation by rat calvaria cells *in vitro*, revealing that IL-6 may inhibit osteoblast differentiation (26). In obesity, elevated level of IL-6 may lead to a low-grade inflammatory state and bone metabolism imbalance, but the mechanism remains unclear.

It was reported that obesity accelerated senescence of BMSCs (including the increased expression of senescence markers p53 and p21) in the BM microenvironment, thereby preventing BMSCs recruitment for bone remodeling and leading to bone fragility (27). IL-6 may be involved in the pathogenesis of senescence (28–33). The combination of IL-6/sIL-6R complex and gp130 activates the JAK/STAT signal transduction pathway and transmits the signal from cell membrane to nucleus, which is critical to cell cycle transition from G1 to S (34). Previously, IL-6/sIL-6R induced premature senescence in normal human fibroblasts through STAT3/p53 pathway, and there was a potential binding site (5′-TTnnnnGA-3′) of p-STAT3 in the p53 promoter region (30, 35). Moreover, IL-6 activated intracellular STAT3/p53/p21 signal transduction and induced senescence of vascular smooth muscle cells (36). However, it is unclear whether IL-6 involved in the senescence of BMSCs in high-fat diet (HFD)-induced obesity. In summary, current knowledge about the roles of IL-6 in differentiation and senescence of BMSCs is limited in HFD-induced obesity. In this study, we used IL-6 gene knockout (IL-6 KO) mice to investigate the effect of IL-6 on bone metabolism and the potential mechanism in HFD-induced obesity. Our study demonstrated that IL-6 KO may inhibit the senescence of BMSCs, thus led to attenuated bone loss.

## Materials and Methods

### Experimental Animals

All animal experiments have been approved by the Institutional Animal Care and Treatment Committee of Sichuan University in China (Permit number: 2020136A) and were carried out in accordance with the approved guidelines. The male wild type (WT) and IL-6 KO mice generated on C57BL/6 background were purchased from Jackson Laboratory (Bar Harbor, ME, USA). Mice were housed in cages at a temperature of 23 ± 1°C with a 12 h light-dark cycle and had free access to food and water. Eight-week-old WT and IL-6 KO mice were randomly divided into standard diet (SD) and HFD groups. The composition of SD and HFD from Beijing HFK Bioscience Corporation was shown in **Table 1**. Four groups of mice were given diet intervention for 12 weeks: (1) WT mice, fed on SD (WT-SD group); (2) WT mice, fed on HFD (WT-HFD group); (3) IL-6 KO mice, fed on SD (IL-6 KO-SD group); (4) IL-6 KO mice, fed on HFD (IL-6 KO-HFD group). Body weight was measured weekly. Mice were fasted for 12 h at the end of the experiment and euthanized under a general anesthesia. Blood was collected from retroorbital vein prior to sacrifice. Enzyme-linked immunosorbent assay (ELISA) kits (Mbbiology biological, Jiangsu, China) were used for detecting levels of IL-6 and procollagen I N-terminal peptide (PINP).

**Table 1:** The composition of standard diet (SD) and high fat diet (HFD).

Composition	SD		HFD	
**Protein**	**19.2 g%**	**20 kcal%**	**26 g%**	**20 kcal%**
Casein	189.58 g	758.32 kcal	258.45 g	1033.80 kcal
Cystine	2.84 g	11.36 kcal	3.88 g	15.52 kcal
**Carbohydrate**	**67.3 g%**	**70 kcal%**	**26 g%**	**20 kcal%**
Corn starch	298.59 g	1194.36 kcal	0	0
Maltodextrin	33.18 g	132.72 kcal	161.53 g	646.12 kcal
Saccharose	331.77 g	1327.08 kcal	88.91 g	355.64 kcal
**Fat**	**4.3 g%**	**10 kcal%**	**35 g%**	**60 kcal%**
Soybean oil	23.70 g	213.30 kcal	32.31 g	290.79 kcal
Lard oil	18.96 g	170.64 kcal	316.60 g	2849.40 kcal
Cellulose	47.40 g	0	64.61 g	0
Others	53.98 g	37.92 kcal	73.71 g	51.68 kcal
Total	1000 g	3845.70 kcal	1000 g	5242.95 kcal

Others: mineral mixture, calcium hydrophosphate, calcium carbonate, potassium citrate, vitamin mixture, etc.

### Analysis of Bone Microstructure

Left femurs and L3 vertebras were isolated from soft tissues and immersed into fixative solution for μCT analysis (37). The high-resolution μCT system (vivaCT80; Scanco Medical, Switzerland) was used to analyze the bone microstructure of trabecular bone of distal femoral metaphysis and L3 vertebra, and cortical bone of femoral mid-diaphysis. The scanner was set at a voltage of 55 kVp, a current of 145 µA and a voxel size of 10 µm. The three-dimensional (3D) reconstruction and analysis were performed using the Scanco software version 5.0. One hundred contiguous cross-sectional slices were selected for analysis of trabecular and cortical bone. The domain of trabecular and cortical analysis was manually profiled and intermediate sections were interpolated with the contouring algorithm to choose a region of interest (ROI). The parameters including bone volume per tissue volume (BV/TV), connect density (Conn. D), structure model index (SMI), trabecular number (Tb. N), trabecular thickness (Tb. Th) and trabecular spacing (Tb. Sp) were achieved for analysis of trabecular bone, while BV/TV, cortical thickness (Ct. Th) and bone surface per bone volume (BS/BV) for cortical bone.

### Quantification and Imaging of BMAT by Osmium-μCT

The BMAT was quantified by osmium tetroxide (osmic acid) staining combined with μCT imaging (Quantum GX, PerkinElmer, USA) (38). The right tibias were dissected from soft tissues, fixed for 24 h, washed for 10 min, and immersed into 20% ethylenediaminetetraacetic acid (EDTA) solution to decalcify at 37°C for 14–17 d. The decalcification solution was changed every 3 d until the bones were flexible. One part 5% solution of potassium dichromate and one part 2% solution of osmium tetroxide were added to a 2-ml microtube, each with 3–4 bones inside. The bones were immersed in the dye for 48 to 60 h at room temperature and washed repeatedly with distilled water for 2 h. The μCT system was used to perform 2D analysis of BMAT in tibias. The scanner was set at a voltage of 90 kVp, a current of 88 µA and a voxel size of 50 µm. The analysis of the osmium density (white color in BM cavity) of tibial sagittal plane was used to assess the content of BMAT with Image J software.

### Histological Analysis

The left tibias were dissected from soft tissues, fixed for 12 h, washed for 10 min, and decalcified at 37°C until the bones were flexible. The tibias were then dehydrated, embedded in paraffin, cut to 5 μm sections, dried, and kept at room temperature. Longitudinal sections from the proximal tibias were stained with either hematoxylin-eosin (H&E) or tartrate-resistant acid phosphatase (TRAP) (Sigma, Merck, Germany). Lipid droplets were counted and calculated to assess BM adiposity using Image J software. TRAP-positive multinucleated cells were observed along the bone edge. Osteoclast surface per bone surface (OcS/BS) was calculated at five different visual fields with Image J software to evaluate osteoclast formation.

### Isolation of Bone Marrow Cells (BMCs)

Both femurs and tibias of WT and IL-6 KO mice were collected and cleaned in PBS. The femoral and tibial diaphysis were snipped and the BMCs were isolated as previously described (39). Bones were placed vertically in a 0.5-ml microtube that was cut open at the bottom. The 0.5-ml microtube was placed into a 1.5-ml microtube. Fresh BM was spun out by quick centrifuge (from 0 to 10,000 rpm within 10 s) at room temperature. Red blood cells were lysed using erythrocyte lysing buffer (Beyotime, Shanghai, China) and the BM suspension was allowed to stand for 5–10 min to make low-density bone marrow adipocytes (BMAs) released and floating. After centrifugation (3,000 rpm for 3 min), the bottom BMCs were collected and washed with PBS for the follow-up experiments. BMCs contained BMSCs, hematopoietic cells, immune cells, etc. but not BMAs or erythrocytes.

### Colony Formation

BMCs harvested from femoral and tibial cavities of WT and IL-6 KO mice after diet intervention were plated at a density of 5 × 10^5^ cells/well in 6-well culture plates (40). BMCs were cultured with MEM alpha modification (αMEM, HyClone, Thermo Fisher Scientific, USA), containing 10% fetal bovine serum (FBS, Gibco, Thermo Fisher Scientific, USA) and 1% penicillin–streptomycin solution. After 24 h of adhesion, nonadherent cells were discarded and the culture medium was changed every other day. Colonies were cultured for 14 d in growth culture medium. Then the medium was removed and cells were stained with 0.1% crystal violet (Beyotime) after fixed with 4% paraformaldehyde solution. Colonies were counted in three different wells with Image J software.

### Replicative Senescent BMSCs

BMCs from WT and IL-6 KO mice were cultured in αMEM, digested and passaged with TrypLE Express Enzyme (Gibco). BMCs were cultured for passage 9 (P9) to achieve replicative senescent BMSCs as described previously (41), with minor modification. BMCs from WT mice were cultured with or without 100 ng/ml mouse IL-6 neutralization antibody (R&D Systems, USA), while cells from IL-6 KO mice were cultured with or without 2 ng/ml recombinant mouse IL-6 (Solarbio, Beijing, China). The medium was changed every other day. BMCs, containing hematopoietic cells, immune cells etc., were purified into BMSCs through culturing and passaging. The senescent BMSCs were used for senescence‐associated‐β‐galactosidase (SA‐β‐gal) staining, and analysis of related mRNA and protein expression.

### SA‐β‐Gal (Senescence-Associated-β-Galactosidase) Staining

SA-β-gal staining of BMSCs was conducted as previously described (42). Senescence β-galactosidase staining kit and X-gal were purchased from Cell Signaling Technology (CST, USA). Briefly, cells were fixed in fix solution at room temperature for 15 min and stained with fresh staining solution at 37°C overnight. SA-β-gal-positive cells were counted in randomly selected five fields by Image J software.

### RNA Extraction, cDNA Synthesis, and Quantitative RT-PCR (qRT-PCR)

Total RNA was extracted from the distal metaphysis of right femurs, BMCs and cultured BMSCs according to the protocol provided by the manufacturer with TRIzol reagent (Invitrogen, Thermo Fisher Scientific, USA). The total RNA (1 μg) was converted to cDNA using PrimeScript RT reagent Kit with gDNA Eraser (Takara, Japan). Gene expression analysis was performed using LightCycler 96 Real-Time PCR System (Roche, Switzerland) and TB Green Premix Ex Taq II (Takara). Primer sequences were summarized in **Table 2**. The relative mRNA levels of target genes were normalized to that of β-actin. Data analysis was performed with the 2^-ΔΔCT^ method.

**Table 2:** Primer sequences.

Target gene	Forward (5′ to 3′)	Reverse (5′ to 3′)
β-actin	AGATTACTGCTCTGGCTCCTAGC	ACTCATCGTACTCCTGCTTGCT
Col1a1	CTGGCGGTTCAGGTCCAAT	TTCCAGGCAATCCACGAGC
Col1a2	CCCAGAGTGGAACAGCGATT	ATGAGTTCTTCGCTGGGGTG
Adipoq	ATCTGGAGGTGGGAGACCAA	GGGCTATGGGTAGTTGCAGT
Pparg	CACTCGCATTCCTTTGACATC	CGCACTTTGGTATTCTTGGAG
Lepr	GGTCCTCTTCTTCTGGAGCCT	AGAACTGCTTTCAGGGTCTGG
p53	TCAGCCTCTTGATGACTGCC	ATCGTCCATGCAGTGAGGTG
p21	GTGAGGAGGAGCATGAATGGA	GAACAGGTCGGACATCACCA
p16	CGCTTCTCACCTCGCTTGT	TGACCAAGAACCTGCGACC

### Western Blot Analysis

The total protein from BMCs and cultured BMSCs was obtained by using RIPA lysis buffer with general protease inhibitor cocktail and general phosphatase inhibitor cocktail (Absin, Shanghai, China). Protein lysates were quantified using a BCA quantification kit (Absin), subjected to sodium dodecyl sulfate-polyacrylamide gels (SDS-PAGE) and electrotransferred to PVDF membranes (GE Life Sciences, USA). The membranes were blocked with 5% milk solution, incubated with primary and secondary antibodies in sequence. The primary antibodies against β-actin, p21, p53, STAT3 and phospho-STAT3, as well as secondary antibodies were from CST. The results of western blot analysis were obtained by ChemiDoc XRS+ system (Bio-Rad, USA) with ECL reagents (GE Life Sciences).

### Statistical Analysis

Results were presented as mean ± standard deviation (SD). Statistical analysis was performed using SPSS 5.0 software. Statistically significant differences between two groups were determined using unpaired, two-tailed Student’s *t* test, and two-way ANOVA (genotype x diet) with post-hoc test was used for multiple group comparisons. Actual *P* values have been shown in each graph or statistically significant *p* values were labeled as follows: * *P*<0.05, ** *P*<0.01, *** *P*<0.001 (compared to the same genotype); ^#^ *P*<0.05, ^##^ *P*<0.01, ^###^ *P*<0.001 (compared to the same diet).

## Results

### IL-6 KO Restrained Trabecular Bone Loss in HFD-induced Obesity

**Figure 1:**
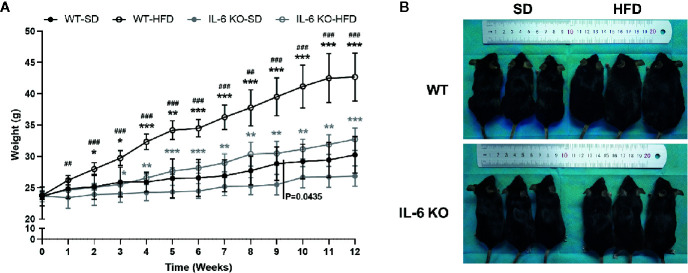
The obesity models were established with WT and IL-6 KO mice. **(A)** Body weight curves of WT and IL-6 KO mice after SD or HFD (n = 5). **(B)** Photographs of mice in each group after 12 weeks of dietary intervention. Data were expressed as mean ± SD, *P < 0.05, **P < 0.01, ***P < 0.001: compared to the same genotype; ^#^ *P* < 0.05, ^##^ *P* < 0.01, ^###^ *P* < 0.001: compared to the same diet.

**Figure 2:**
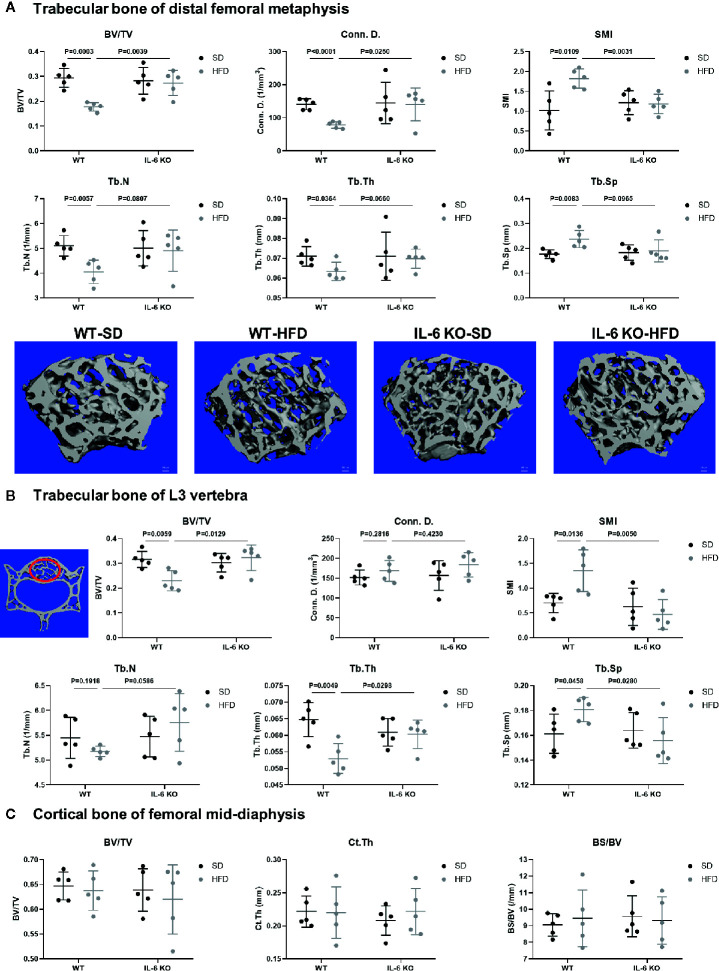
IL-6 KO restrained the trabecular bone loss in HFD-induced obesity. **(A)** The trabecular bone parameters at distal femoral metaphysis were evaluated as BV/TV, Conn. D, SMI, Tb. N, Tb. Th and Tb. Sp from WT and IL-6 KO mice after SD or HFD treatment. Representative images for μCT 3D reconstruction were shown in lower panel (scale bar = 100 μm). **(B)** The trabecular bone parameters at the L3 vertebra were measured *via* μCT from WT and IL-6 KO mice after SD or HFD treatment. The red circles indicate the ROI. **(C)** The cortical bone parameters at femoral mid-diaphysis measurement were evaluated as BV/TV, Ct. Th and BS/BV from WT and IL-6 KO mice after SD or HFD treatment. Data were expressed as mean ± SD.

The obesity models were established with WT and IL-6 KO mice after HFD for 12 weeks. Compared with the SD, the weight of WT mice was increased by 25%, 36%, and 41% after HFD for 4, 8, and 12 weeks, while the weight of IL-6 KO mice was increased by 9%, 20%, and 22% (**Figures 1A, B**). Moreover, we found that the levels of IL-6 in serum increased significantly after HFD in WT mice (**Table 3**). To evaluate the effects of HFD and IL-6 on the bone mass and bone microstructure, μCT was used to assay the trabecular bone of distal femoral metaphysis, L3 vertebra, and the cortical bone of femoral mid-diaphysis. On the SD, no difference was observed between WT and IL-6 KO mice in the trabecular bone of the distal femoral metaphysis (**Figure 2A**) and the L3 vertebra (**Figure 2B**). On the HFD, the distal femoral metaphysis of WT mice showed obvious reduction in trabecular BV/TV, Conn. D, Tb. N and Tb. Th, and prominent increase in SMI and Tb. Sp, the L3 vertebra of WT mice showed distinct reduction in trabecular BV/TV and Tb. Th, and significant increase in SMI and Tb. Sp. The distal femoral metaphysis and L3 vertebra of IL-6 KO mice showed no significant changes in bone mass and bone microstructure after HFD. Additionally, no significant changes were detected in cortical BV/TV, Ct. Th and BS/BV in the femoral mid-diaphysis of WT and IL-6 KO mice after HFD (**Figure 2C**). These results indicated that IL-6 KO retained trabecular bone loss in HFD-induced obesity.

**Table 3:** The levels of IL-6 in serum and culture medium of BMSCs in WT mice after SD or HFD.

Mice	Serum (n = 7)	Culture medium of primary BMSCs (n = 8)
WT-SD WT-HFD	15.2 ± 6.4 pg/ml 28.9 ± 7.1 pg/ml **	11.0 ± 1.0 pg/ml 20.8 ± 2.5 pg/ml ***

Data were expressed as mean ± SD. **P < 0.01, ***P < 0.001: WT-HFD mice versus WT-SD mice.

### IL-6 KO Rescued the Decreased Osteogenesis in HFD-induced Obesity

**Figure 3:**
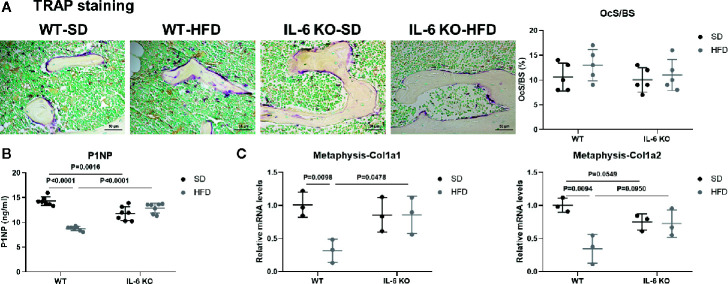
IL-6 KO rescued the decreased osteogenesis in HFD-induced obesity. **(A)** Histopathological analysis on bone sections from tibia stained with TRAP staining from WT and IL-6 KO mice after SD or HFD treatment (scale bar = 50 μm). Osteoclast surface per bone surface (OcS/BS) was evaluated in tibia (right panel). **(B)** Bone formation marker PINP was measured in serum of WT and IL-6 KO mice after SD or HFD. **(C)** The mRNA levels of osteogenic markers Col1a1 and Col1a2 were measured in metaphysis of WT and IL-6 KO mice after SD or HFD. Data were expressed as mean ± SD.

To investigate the possible contributor of bone loss after HFD, we further analyzed the bone turnover indicators. TRAP staining of proximal tibia showed no significant difference between WT and IL-6 KO mice after SD or HFD (**Figure 3A**). The level of serum bone formation biomarker PINP was significantly decreased in WT mice (14.3 to 8.7 ng/ml) after HFD, while it kept a similar level in IL-6 KO mice (11.8 to 12.9 ng/ml) after HFD (**Figure 3B**). In addition, mRNA levels of collagen type 1 alpha 1 chain (Col1a1) and Col1a2 in metaphysis of WT mice were significantly reduced, while those in IL-6 KO mice did not show notable changes after HFD (**Figure 3C**). These results suggested that HFD inhibited osteogenic differentiation, while IL-6 KO rescued the HFD-induced decreased osteogenesis.

### IL-6 KO Attenuated Adipogenesis of BM in HFD-induced Obesity

**Figure 4:**
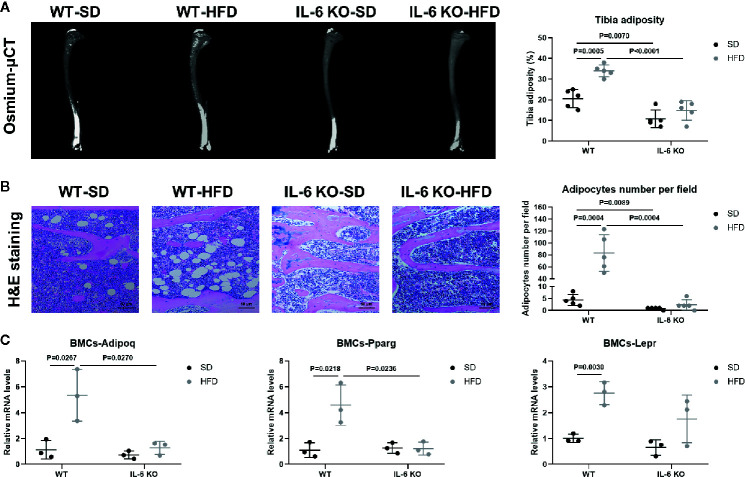
IL-6 KO attenuated adipogenesis of BM in HFD-induced obesity. **(A)** Representative images of BMAT from full-length tibial sagittal plane of WT and IL-6 KO mice fed with SD or HFD by μCT. Quantification of the osmium density in tibial sagittal plane was expressed as tibia adiposity (right panel). **(B)** Histopathological analysis on bone sections from tibia stained with H&E staining from WT and IL-6 KO mice after SD or HFD treatment (scale bar = 50 μm). Adipocyte number per field on H&E section was evaluated by Image J software (right panel). **(C)** The mRNA levels of adipogenic genes in bone marrow cells (BMCs) of WT and IL-6 KO mice after SD or HFD. Data were expressed as mean ± SD.

HFD induced significant obesity in mice of both strains, while the weight gain of IL-6 KO-HFD mice was significantly lower than that of WT-HFD mice. Obesity-induced osteoporosis is closely related to osteogenesis and adipogenesis in the BM. We next investigated BM adiposity *via* osmium-μCT and H&E staining. On the SD, IL-6 KO mice exhibited less BMAT, compared with WT mice (**Figure 4A**). The osmium signal was increased in WT mice, while it was not statistically changed in IL-6 KO mice after HFD. In addition, the amounts of adipocytes in proximal tibia by H&E staining were statistically increased only in WT mice, but not in IL-6 KO mice (**Figure 4B**). These data indicated that HFD induced a greater amount of BMAT in WT mice than that in IL-6 KO mice.

Since the osteogenesis and adipogenesis of BMSCs created a tug-of-war between osteoblasts and adipocytes, we next examined the mRNA levels of adipogenic differentiation genes in bone marrow cells (BMCs). BMCs contained BMSCs, hematopoietic cells, immune cells, etc. but not BMAs or erythrocytes. The BMCs from WT-HFD mice exhibited increased adipocytic differentiation capacity compared with WT-SD mice measured by mRNA gene expression of adipogenic genes (Adipoq, Pparg, Lepr), indicating that HFD increased an adipogenic cell population in BM (**Figure 4C**). However, the adipogenic gene expression (Adipoq, Pparg) in the BMCs from IL-6 KO mice was not significantly changed after HFD, which suggested that IL-6 KO restrained HFD-induced BMAT expansion. These results implied that IL-6 might enhance the adipogenic potential of BMSCs to accelerate trabecular bone loss in HFD-induced obesity.

### IL-6 KO Attenuated Senescence of BMCs in HFD-induced Bone Loss

**Figure 5:**
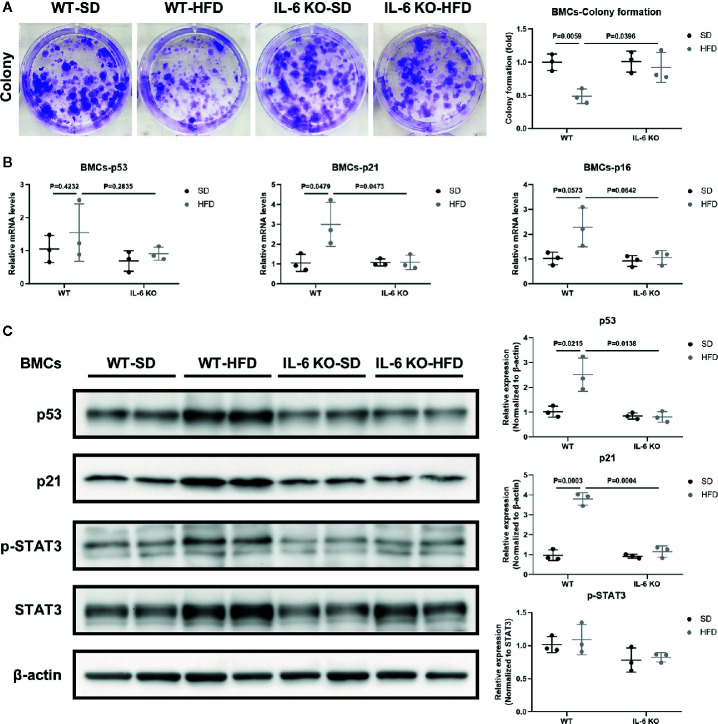
IL-6 KO attenuated senescence of BMCs in HFD-induced bone loss. **(A)** Representative images for colony formation of bone marrow cells (BMCs) from WT and IL-6 KO mice after SD or HFD treatment. The number of colonies per well was counted and calculated (right panel). **(B)** The mRNA levels of typical aging markers (p53, p21, p16) in BMCs from WT and IL-6 KO mice after SD or HFD. **(C)** Western blot analysis of indicated proteins (p53, p21, p-STAT3, STAT3, β-actin) in BMCs of WT and IL-6 KO mice fed with SD or HFD for 12 weeks. The expression of p53 and p21 was normalized against β-actin, and the expression of p-STAT3 was normalized against STAT3. Quantitative analysis with Image J software was shown on the right panel. Data were expressed as mean ± SD.

We have observed the differences in the increase of adipogenesis and the decrease of osteogenesis in BM of the two strains of mice in HFD-induced obesity, and obesity can also accelerate cellular senescence (27, 43, 44). As an important component of senescence-associated secretory phenotype (SASP), IL-6 can enhance cell senescence through autocrine and paracrine pathways (45). The HFD induced distinct increase in IL-6 levels of the culture supernatant of primary BMSCs in WT mice (**Table 3**), suggesting the senescent phenotype of BMSCs. Considering the senescence of BMSCs promoted bone loss (27), we explored whether IL-6 KO could inhibit senescence of BMSCs. Bone marrow cells (BMCs), containing hematopoietic cells, immune cells etc., were purified into BMSCs through culturing. The number of colonies from BMCs was significantly decreased in WT mice after HFD, while it remained similarly in IL-6 KO mice after HFD (**Figure 5A**). The changes in mRNA levels of typical senescence marker p16 were not statistically significant in the four groups of mice (**Figure 5B**). However, p21, another typical aging marker, in BMCs was significantly increased in WT-HFD mice while it did not change observably in IL-6 KO-HFD mice (**Figures 5B, C** **)**. Although the mRNA level of p53 in WT mice showed only a slight increase after HFD, the protein level was significantly increased. Moreover, the protein level of p53 in BMCs was not significantly changed in IL-6 KO mice after HFD. It was reported that IL-6 activated intracellular STAT3/p53/p21 signal transduction and induced senescence of vascular smooth muscle cells (36). As IL-6/sIL-6R was involved in the pathogenesis of cell senescence through IL-6/sIL-6R/STAT3 axis (30, 32–34), we explored this pathway in the process of senescence in BMCs. HFD induced a similar protein level of p-STAT3 in WT and IL-6 KO mice (**Figure 5C**). These results suggested IL-6 KO attenuated the reduced proliferation of BMCs and the enhanced expression of senescence markers in HFD-induced bone loss, which implied that IL-6 promoted senescence of BMCs.

### IL-6 Might Accelerate Senescence of BMSCs through IL-6/STAT3 Pathway

**Figure 6:**
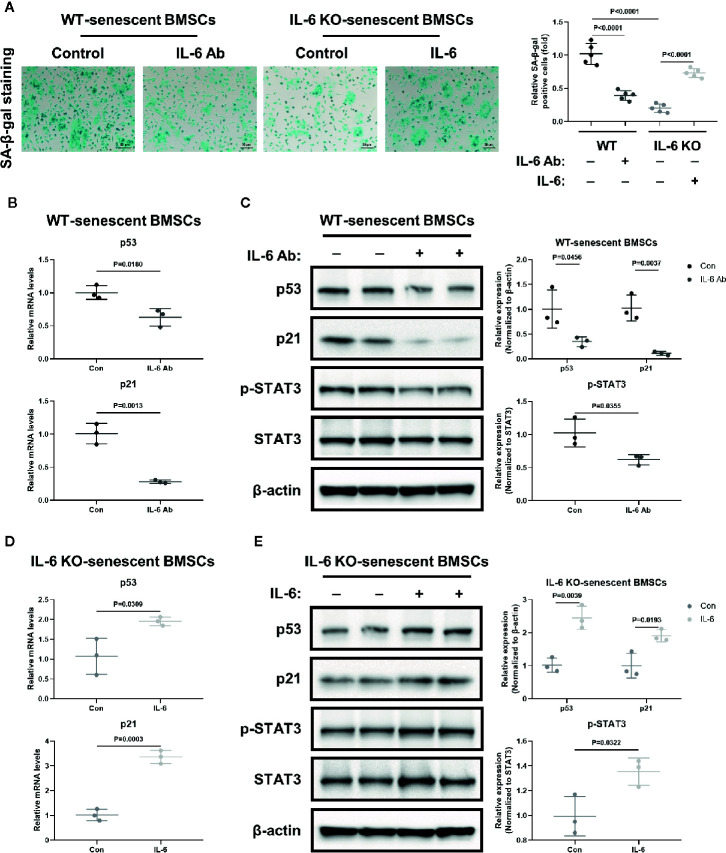
IL-6 accelerated senescence of BMSCs through IL-6/STAT3 pathway. **(A)** Representative images for SA-β-Gal staining of replicative BMSCs with or without IL-6 neutralization antibody from WT mice, and with or without recombinant mouse IL-6 treatment from IL-6 KO mice. (Cell nucleus: dark blue, SA-β-gal-positive cell: cyan, scale bar = 50 μm). Quantification of SA-β-Gal positive cells evaluated with Image J software (right panel). **(B)** The mRNA levels of p53 and p21 in replicative BMSCs with or without IL-6 antibody treatment from WT mice. **(C)** Western blot analysis of indicated proteins (p53, p21, p-STAT3, STAT3, β-actin) in replicative BMSCs with or without IL-6 antibody treatment from WT mice. The expression of p53 and p21 was normalized against β-actin, and the expression of p-STAT3 was normalized against STAT3. Quantitative analysis with Image J software was shown on the right panel. **(D)** The mRNA levels of p53 and p21 in replicative BMSCs with or without IL-6 treatment from IL-6 KO mice. **(E)** Western blot analysis of indicated proteins in replicative BMSCs with or without recombinant IL-6 treatment from IL-6 KO mice. The expression of p53 and p21 was normalized against β-actin, and the expression of p-STAT3 was normalized against STAT3. Quantitative analysis with Image J software was shown on the lower panel. Data were expressed as mean ± SD.

BMSCs are the common ancestor of osteoblasts and adipocytes. The status of BMSCs accurately reflects the potential of adipogenic and osteogenic differentiation. To further investigate the role of IL-6 on BMSCs senescence, we generated replicative senescent BMSCs from BMCs of WT and IL-6 KO mice by cultivation and passage. SA-β-gal is a key feature in the process of cell senescence. The SA-β-gal positive BMSCs from IL-6 KO mice were significantly less than that from WT mice when cultured for passage 9 (P9) (**Figure 6A**). IL-6 neutralization antibody exposure resulted in significant reduction in SA-β-gal positive cells in WT mice, while BMSCs from IL-6 KO mice showed more positive cells after being treated with recombinant IL-6. Furthermore, the mRNA and protein levels of senescence markers (p53, p21) in BMSCs from WT were markedly decreased after IL-6 antibody treatment (**Figures 6B, C**). In contrast, the mRNA and protein levels of senescence markers (p53, p21) in BMSCs from IL-6 KO mice were significantly increased after recombinant IL-6 treatment (**Figures 6D, E**). The protein level of p-STAT3 was notably decreased in senescent BMSCs from WT mice with IL-6 antibody treatment, while expression of p-STAT3 showed significant increase in BMSCs from IL-6 KO mice after recombinant IL-6 treatment (**Figures 6C, E**). Taken together, these results implied that IL-6 accelerated senescent phenotype of BMSCs through the IL-6/STAT3 pathway, suggesting a potential mechanism for bone loss in HFD-induced obesity.

## Discussion

The body weight of WT and IL-6 KO mice was increased significantly after HFD, while IL-6 KO mice gained less weight than WT mice (**Figures 1A, B**). The changes of body weight were consistent with the previous study (46), but not exactly as the same as the results of several other studies due to the different dietary intervention time, the consistency of the initial body weight of the two strains of mice, and the animal conditions (47–49). Previous study has suggested that the trabecular BV/TV, Tb. N and Tb. Th of IL-6 KO mice were higher than those of WT mice through bone histomorphormetry after SD (47). In our study, the high-resolution μCT system was used to analyze the bone microstructure of WT and IL-6 KO mice, and we found similar levels of BV/TV, Conn. D, SMI, Tb. N, Tb. Th and Tb. Sp between WT-SD and IL-6 KO-SD mice. The dietary intervention time and detection methods in our study were different from the previous study (47), which may help explain the inconsistent results of bone phenotypes. In the present study, we found 12-week HFD treatment obviously enhanced trabecular bone loss in WT mice, but it failed to do so in IL-6 KO mice (**Figures 2A, B**).

Evidence has shown that HFD led to chronic low-grade inflammation and local lipid accumulation (50). There are two main imbalances in the development of osteoporosis (OP): one is bone-fat imbalance, the other lies on the uncoupled bone remodeling (9). Bone resorption marker TRAP staining showed no significant difference between WT and IL-6 KO mice after SD or HFD (**Figure 3A**). Furthermore, both the levels of PINP in serum and the mRNA levels of osteogenic markers in metaphysis indicated that IL-6 KO restrained HFD-induced decrease of osteogenesis (**Figures 3B, C**). It was widely accepted that abnormal expansion of BMAT plays a crucial role in the onset and progression of OP, in part because both adipocytes and osteoblasts originate from a common ancestor lineage and there is a competitive relationship between adipogenic and osteogenic differentiation of BMSCs (51). Marrow adipocytes are dynamic: their size and number can change in response to environmental, nutritional, and hormonal cues (39). In our study, IL-6 KO suppressed HFD-induced BMAT expansion (**Figures 4A, B**). In addition, IL-6 KO restrained the increase of adipogenic genes (Adipoq, Pparg) in BMCs induced by HFD (**Figure 4C**). Therefore, IL-6 KO inhibited the adipogenesis in BM, arrested the shift of BMSCs from the osteoblast lineage to the adipocyte lineage.

BM adiposity is a manifestation of BMSCs senescence, and obesity accelerates cell senescence (27, 43, 44). Tencerova *et al.* found BMAT accumulation and BMSCs senescence were increased in BM cavity in obesity (27, 43). Moreover, obesity results in the accumulation of senescent glial cells in proximity to the lateral ventricle, while senescent glial cells exhibit excessive fat deposits (44). As one of the most important inflammatory mediators in obesity, IL-6 was also involved in the pathogenesis of cell senescence. IL-6 KO inhibited aging-related accumulation of p53 in mouse myocardium (52). Cell senescence involves in multiple signaling pathways (53). The senescence associated gene p21 is a well-known target gene of p53 that has been shown to play a critical role during the process of p53 induced cellular senescence (54). We hypothesized that IL-6 KO may restrain HFD-induced accelerated senescent phenotype in the BM microenvironment. Several lines of experimental evidence supported this hypothesis. First, IL-6 KO inhibited the HFD-induced decrease in the number of colonies from BMCs (**Figure 5A**). Secondly, IL-6 KO inhibited HFD-induced increase of senescence-specific markers in BMCs (**Figures 5B, C**). This may explain the possible molecular mechanism of IL-6 KO preventing HFD-induced bone loss. IL-6 KO may prevent HFD-induced BMSCs exhaustion and the creation of a senescent BM microenvironment, thereby relieving bone fragility in HFD-induced obesity.

Senescent cells secrete multiple inflammatory factors, chemokines and matrix proteases, known as senescence-associated secretory phenotype (SASP). IL-6 is an important component of SASP. IL-6 can enhance cell senescence through autocrine and paracrine pathways (45). Replicative BMSCs derived from WT mice when grown in medium supplemented with IL-6 antibody, exhibited consistently less SA-β-gal staining and lower levels of senescent markers (p53, p21) than controls (**Figures 6A–C**). The recombinant IL-6 administration led to increased SA-β-gal staining and higher levels of senescent markers (p53, p21) in replicative BMSCs from the IL-6 KO mice, when compared to controls (**Figures 6A, D, E**).

It was reported that the IL-6/sIL-6R complex activates the JAK/STAT3 signal transduction pathway and inhibits G1 to S phase transition of cell cycle (34, 36, 52). Previously, IL-6/sIL-6R induced premature senescence in normal human fibroblasts through STAT3/p53 pathway, and there was a potential binding site (5′-TTnnnnGA-3′) of p-STAT3 in the p53 promoter region (30, 35). Moreover, IL-6 activated intracellular STAT3/p53/p21 signal transduction and induced senescence of vascular smooth muscle cells (36). In our study, although BMCs from IL-6 KO mice expressed similar levels of p-STAT3 with WT mice after HFD (**Figure 5C**), less p-STAT3 expression was detected when the effects of IL-6 were removed in replicative senescent model using BMSCs from WT and IL-6 KO mice (**Figures 6C, E**). This supports the hypothesis that the activation of IL-6/STAT3 promotes the senescence of BMSCs in BM, suggesting a potential mechanism for trabecular bone loss in HFD-induced obesity.

In summary, we demonstrated the effect of IL-6 on differentiation and senescence of BMSCs in HFD-induced obesity. IL-6 KO restrained HFD-induced trabecular bone loss and BMAT increase. IL-6 might promote BMAT expansion and break the balance between osteogenesis and adipogenesis of BMSCs in HFD-induced bone fragility. IL-6 promoted BMSCs senescence through IL-6/STAT3 pathway, suggesting a potential mechanism for bone loss. Therefore, IL-6 KO contributed to the maintenance of bone mass after HFD. Our results showed, for the first time, that the IL-6 may be involved in the potential mechanism of HFD-induced trabecular bone loss by breaking the balance between osteogenesis and adipogenesis of BMSCs and promoting senescence of BMSCs in HFD-induced obesity.

## Data Availability Statement

The original contributions presented in the study are included in the article/supplementary material. Further inquiries can be directed to the corresponding author.

## Ethics Statement

The animal study was reviewed and approved by the Institutional Animal Care and Treatment Committee of Sichuan University.

## Author Contributions

XY designed this research. YJL, LL, YX, XC, LT, YaL, HL, and JZ were responsible for the experiments. Among them, YJL, LL, YX, XC, and HL were in charge of the animal experiments, cellular experiments, and molecular experiments. YaL and JZ were mainly responsible for the histopathological part. YJL, LT, YiL, and XY were responsible for the revision of the whole article. All authors contributed to the article and approved the submitted version.

## Funding

This work was supported by grants from the National Natural Science Foundation of China (no. 81770875, 81902246), the Project of Health Commission of Sichuan Province (no. 19PJ096), 1.3.5 Project for Disciplines of Excellence of West China Hospital (no. 2020HXFH008, ZYJC18003), and the Post-Doctor Research Project of West China Hospital (no. 19HXBH053).

## Conflict of Interest

The authors declare that the research was conducted in the absence of any commercial or financial relationships that could be construed as a potential conflict of interest.

## References

[1] Kahn SE Hull RL Utzschneider KM. Mechanisms linking obesity to insulin resistance and type 2 diabetes. Nature (2006) 444:840–6. 10.1038/nature05482 17167471

[2] Willebrords J Pereira IV Maes M Crespo Yanguas S Colle I Van Den Bossche B. Strategies, models and biomarkers in experimental non-alcoholic fatty liver disease research. Prog Lipid Res (2015) 59:106–25. 10.1016/j.plipres.2015.05.002 PMC4596006 26073454

[3] Mpalaris V Anagnostis P Goulis DG Iakovou I. Complex association between body weight and fracture risk in postmenopausal women. Obes Rev (2015) 16:225–33. 10.1111/obr.12244 25586664

[4] Nih Consensus Development Panel on Osteoporosis Prevention D. Therapy. Osteoporosis prevention, diagnosis, and therapy. JAMA (2001) 285:785–95. 10.1001/jama.285.6.785 11176917

[5] Evans AL Paggiosi MA Eastell R Walsh JS. Bone density, microstructure and strength in obese and normal weight men and women in younger and older adulthood. J Bone Miner Res (2015) 30:920–8. 10.1002/jbmr.2407 25400253

[6] Cohen A Dempster DW Recker RR Lappe JM Zhou H Zwahlen A. Abdominal fat is associated with lower bone formation and inferior bone quality in healthy premenopausal women: a transiliac bone biopsy study. J Clin Endocrinol Metab (2013) 98:2562–72. 10.1210/jc.2013-1047 PMC3667251 23515452

[7] Compston JE Flahive J Hooven FH Anderson FA Jr. Adachi JD Boonen S. Obesity, health-care utilization, and health-related quality of life after fracture in postmenopausal women: Global Longitudinal Study of Osteoporosis in Women (GLOW). Calcif Tissue Int (2014) 94:223–31. 10.1007/s00223-013-9801-z PMC3917823 24077896

[8] Lecka-Czernik B Stechschulte LA Czernik PJ Dowling AR. High bone mass in adult mice with diet-induced obesity results from a combination of initial increase in bone mass followed by attenuation in bone formation; implications for high bone mass and decreased bone quality in obesity. Mol Cell Endocrinol (2015) 410:35–41. 10.1016/j.mce.2015.01.001 25576855

[9] Li J Chen X Lu L Yu X. The relationship between bone marrow adipose tissue and bone metabolism in postmenopausal osteoporosis. Cytokine Growth Factor Rev (2020) 52:88–98. 10.1016/j.cytogfr.2020.02.003 32081538

[10] Tencerova M Kassem M. The Bone Marrow-Derived Stromal Cells: Commitment and Regulation of Adipogenesis. Front Endocrinol (Lausanne) (2016) 7:127. 10.3389/fendo.2016.00127 27708616 PMC5030474

[11] Abdallah BM Kassem M. New factors controlling the balance between osteoblastogenesis and adipogenesis. Bone (2012) 50:540–5. 10.1016/j.bone.2011.06.030 21745614

[12] Jafari A Qanie D Andersen TL Zhang Y Chen L Postert B. Legumain Regulates Differentiation Fate of Human Bone Marrow Stromal Cells and Is Altered in Postmenopausal Osteoporosis. Stem Cell Rep (2017) 8:373–86. 10.1016/j.stemcr.2017.01.003 PMC5312427 28162997

[13] Veldhuis-Vlug AG Rosen CJ. Clinical implications of bone marrow adiposity. J Intern Med (2018) 283:121–39. 10.1111/joim.12718 PMC5847297 29211319

[14] Amano SU Cohen JL Vangala P Tencerova M Nicoloro SM Yawe JC. Local proliferation of macrophages contributes to obesity-associated adipose tissue inflammation. Cell Metab (2014) 19:162–71. 10.1016/j.cmet.2013.11.017 PMC3931314 24374218

[15] McLean RR. Proinflammatory cytokines and osteoporosis. Curr Osteoporos Rep (2009) 7:134–9. 10.1007/s11914-009-0023-2 19968917

[16] Schett G. Effects of inflammatory and anti-inflammatory cytokines on the bone. Eur J Clin Invest (2011) 41:1361–6. 10.1111/j.1365-2362.2011.02545.x 21615394

[17] Souza PP Lerner UH. The role of cytokines in inflammatory bone loss. Immunol Invest (2013) 42:555–622. 10.3109/08820139.2013.822766 24004059

[18] Mohamed-Ali V Goodrick S Rawesh A Katz DR Miles JM Yudkin JS. Subcutaneous adipose tissue releases interleukin-6, but not tumor necrosis factor-alpha, in vivo. J Clin Endocrinol Metab (1997) 82:4196–200. 10.1210/jcem.82.12.4450 9398739

[19] Tchkonia T Morbeck DE Von Zglinicki T Van Deursen J Lustgarten J Scrable H. Fat tissue, aging, and cellular senescence. Aging Cell (2010) 9:667–84. 10.1111/j.1474-9726.2010.00608.x PMC2941545 20701600

[20] Schaper F Rose-John S. Interleukin-6: Biology, signaling and strategies of blockade. Cytokine Growth Factor Rev (2015) 26:475–87. 10.1016/j.cytogfr.2015.07.004 26189695

[21] Sims NA Jenkins BJ Quinn JM Nakamura A Glatt M Gillespie MT. Glycoprotein 130 regulates bone turnover and bone size by distinct downstream signaling pathways. J Clin Invest (2004) 113:379–89. 10.1172/JCI19872 PMC324544 14755335

[22] Xie Z Tang S Ye G Wang P Li J Liu W. Interleukin-6/interleukin-6 receptor complex promotes osteogenic differentiation of bone marrow-derived mesenchymal stem cells. Stem Cell Res Ther (2018) 9:13. 10.1186/s13287-017-0766-0 29357923 PMC5776773

[23] Bellido T Borba VZ Roberson P Manolagas SC. Activation of the Janus kinase/STAT (signal transducer and activator of transcription) signal transduction pathway by interleukin-6-type cytokines promotes osteoblast differentiation. Endocrinology (1997) 138:3666–76. 10.1210/endo.138.9.5364 9275051

[24] Nishimura R Moriyama K Yasukawa K Mundy GR Yoneda T. Combination of interleukin-6 and soluble interleukin-6 receptors induces differentiation and activation of JAK-STAT and MAP kinase pathways in MG-63 human osteoblastic cells. J Bone Miner Res (1998) 13:777–85. 10.1359/jbmr.1998.13.5.777 9610741

[25] Roodman GD. Interleukin-6: an osteotropic factor? J Bone Miner Res (1992) 7:475–8. 10.1002/jbmr.5650070502 1615755

[26] Hughes FJ Howells GL. Interleukin-6 inhibits bone formation in vitro. Bone Miner (1993) 21:21–8. 10.1016/s0169-6009(08)80117-1 8324417

[27] Tencerova M Frost M Figeac F Nielsen TK Ali D Lauterlein JL. Obesity-Associated Hypermetabolism and Accelerated Senescence of Bone Marrow Stromal Stem Cells Suggest a Potential Mechanism for Bone Fragility. Cell Rep (2019) 27:2050–62.e6. 10.1016/j.celrep.2019.04.066 31091445

[28] Ershler WB. Interleukin-6: a cytokine for gerontologists. J Am Geriatr Soc (1993) 41:176–81. 10.1111/j.1532-5415.1993.tb02054.x 8426042

[29] Erol A. Interleukin-6 (IL-6) is still the leading biomarker of the metabolic and aging related disorders. Med Hypotheses (2007) 69:708. 10.1016/j.mehy.2007.01.021 17335991

[30] Kojima H Kunimoto H Inoue T Nakajima K. The STAT3-IGFBP5 axis is critical for IL-6/gp130-induced premature senescence in human fibroblasts. Cell Cycle (2012) 11:730–9. 10.4161/cc.11.4.19172 22374671

[31] Kuilman T Michaloglou C Vredeveld LC Douma S van Doorn R Desmet CJ. Oncogene-induced senescence relayed by an interleukin-dependent inflammatory network. Cell (2008) 133:1019–31. 10.1016/j.cell.2008.03.039 18555778

[32] Ren C Cheng X Lu B Yang G. Activation of interleukin-6/signal transducer and activator of transcription 3 by human papillomavirus early proteins 6 induces fibroblast senescence to promote cervical tumorigenesis through autocrine and paracrine pathways in tumor microenvironment. Eur J Cancer (2013) 49:3889–99. 10.1016/j.ejca.2013.07.140 23953057

[33] Hodge DR Peng B Cherry JC Hurt EM Fox SD Kelley JA. Interleukin 6 supports the maintenance of p53 tumor suppressor gene promoter methylation. Cancer Res (2005) 65:4673–82. 10.1158/0008-5472.CAN-04-3589 15930285

[34] Hirano T Ishihara K Hibi M. Roles of STAT3 in mediating the cell growth, differentiation and survival signals relayed through the IL-6 family of cytokine receptors. Oncogene (2000) 19:2548–56. 10.1038/sj.onc.1203551 10851053

[35] Kojima H Inoue T Kunimoto H Nakajima K. IL-6-STAT3 signaling and premature senescence. JAKSTAT (2013) 2:e25763. 10.4161/jkst.25763 24416650 PMC3876432

[36] Xu D Zeng F Han L Wang J Yin Z Lv L. The synergistic action of phosphate and interleukin-6 enhances senescence-associated calcification in vascular smooth muscle cells depending on p53. Mech Ageing Dev (2019) 182:111124. 10.1016/j.mad.2019.111124 31376399

[37] Beamer WG Shultz KL Ackert-Bicknell CL Horton LG Delahunty KM Coombs HF 3rd. Genetic dissection of mouse distal chromosome 1 reveals three linked BMD QTLs with sex-dependent regulation of bone phenotypes. J Bone Miner Res (2007) 22:1187–96. 10.1359/jbmr.070419 17451375

[38] Scheller EL Troiano N Vanhoutan JN Bouxsein MA Fretz JA Xi Y. Use of osmium tetroxide staining with microcomputerized tomography to visualize and quantify bone marrow adipose tissue in vivo. Methods Enzymol (2014) 537:123–39. 10.1016/B978-0-12-411619-1.00007-0 PMC4097010 24480344

[39] Fan Y Hanai JI Le PT Bi R Maridas D DeMambro V. Parathyroid Hormone Directs Bone Marrow Mesenchymal Cell Fate. Cell Metab (2017) 25:661–72. 10.1016/j.cmet.2017.01.001 PMC5342925 28162969

[40] Sun P Jia K Zheng C Zhu X Li J He L. Loss of Lgr4 inhibits differentiation, migration and apoptosis, and promotes proliferation in bone mesenchymal stem cells. J Cell Physiol (2019) 234:10855–67. 10.1002/jcp.27927 30536377

[41] Deng L Ren R Liu Z Song M Li J Wu Z. Stabilizing heterochromatin by DGCR8 alleviates senescence and osteoarthritis. Nat Commun (2019) 10:3329. 10.1038/s41467-019-10831-8 31350386 PMC6659673

[42] Debacq-Chainiaux F Erusalimsky JD Campisi J Toussaint O. Protocols to detect senescence-associated beta-galactosidase (SA-betagal) activity, a biomarker of senescent cells in culture and in vivo. Nat Protoc (2009) 4:1798–806. 10.1038/nprot.2009.191 20010931

[43] Tencerova M Figeac F Ditzel N Taipaleenmaki H Nielsen TK Kassem M. High-Fat Diet-Induced Obesity Promotes Expansion of Bone Marrow Adipose Tissue and Impairs Skeletal Stem Cell Functions in Mice. J Bone Miner Res (2018) 33:1154–65. 10.1002/jbmr.3408 29444341

[44] Ogrodnik M Zhu Y Langhi LGP Tchkonia T Kruger P Fielder E. Obesity-Induced Cellular Senescence Drives Anxiety and Impairs Neurogenesis. Cell Metab (2019) 29:1061–77.e8. 10.1016/j.cmet.2018.12.008 30612898 PMC6509403

[45] Mosteiro L Pantoja C de Martino A Serrano M. Senescence promotes in vivo reprogramming through p16(INK)(4a) and IL-6. Aging Cell (2018) 17:e12711. 10.1111/acel.12711 PMC5847859 29280266

[46] Chen F Chen D Zhao X Yang S Li Z Sanchis D. Interleukin-6 deficiency facilitates myocardial dysfunction during high fat diet-induced obesity by promoting lipotoxicity and inflammation. Biochim Biophys Acta Mol Basis Dis (2017) 1863:3128–41. 10.1016/j.bbadis.2017.08.022 28844956

[47] Feng W Liu B Liu D Hasegawa T Wang W Han X. Long-Term Administration of High-Fat Diet Corrects Abnormal Bone Remodeling in the Tibiae of Interleukin-6-Deficient Mice. J Histochem Cytochem (2016) 64:42–53. 10.1369/0022155415611931 26416243 PMC4810790

[48] Wang C Tian L Zhang K Chen Y Chen X Xie Y. Interleukin-6 gene knockout antagonizes high-fat-induced trabecular bone loss. J Mol Endocrinol (2016) 57:161–70. 10.1530/JME-16-0076 27493246

[49] Chen X Gong Q Wang CY Zhang K Ji X Chen YX. High-Fat Diet Induces Distinct Metabolic Response in Interleukin-6 and Tumor Necrosis Factor-alpha Knockout Mice. J Interferon Cytokine Res (2016) 36:580–8. 10.1089/jir.2016.0022 27610743

[50] Airaksinen K Jokkala J Ahonen I Auriola S Kolehmainen M Hanhineva K. High-Fat Diet, Betaine, and Polydextrose Induce Changes in Adipose Tissue Inflammation and Metabolism in C57BL/6J Mice. Mol Nutr Food Res (2018) 62:e1800455. 10.1002/mnfr.201800455 30290084

[51] Song L Liu M Ono N Bringhurst FR Kronenberg HM Guo J. Loss of wnt/beta-catenin signaling causes cell fate shift of preosteoblasts from osteoblasts to adipocytes. J Bone Miner Res (2012) 27:2344–58. 10.1002/jbmr.1694 PMC3474875 22729939

[52] Bonda TA Dziemidowicz M Cieslinska M Tarasiuk E Wawrusiewicz-Kurylonek N Bialuk I. Interleukin 6 Knockout Inhibits Aging-Related Accumulation of p53 in the Mouse Myocardium. J Gerontol A Biol Sci Med Sci (2019) 74:176–82. 10.1093/gerona/gly105 29718116

[53] Kim YY Jee HJ Um JH Kim YM Bae SS Yun J. Cooperation between p21 and Akt is required for p53-dependent cellular senescence. Aging Cell (2017) 16:1094–103. 10.1111/acel.12639 PMC5595696 28691365

[54] Brugarolas J Chandrasekaran C Gordon JI Beach D Jacks T Hannon GJ. Radiation-induced cell cycle arrest compromised by p21 deficiency. Nature (1995) 377:552–7. 10.1038/377552a0 7566157

